# *Sulawesimetopushenryi*, a new genus and species of Isometopinae (Hemiptera, Heteroptera, Miridae) from Sulawesi

**DOI:** 10.3897/zookeys.796.21273

**Published:** 2018-11-15

**Authors:** Aleksander Herczek, Jacek Gorczyca, Artur Taszakowski

**Affiliations:** 1 Silesian University, Department of Zoology, 40-Katowice, Bankowa 9, Poland Silesian University Katowice Poland

**Keywords:** Heteroptera, Miridae, Isometopinae, *
Sulawesimetopus
*, Indonesia

## Abstract

A new genus and species, *Sulawesimetopushenryi* Herczek, Gorczyca & Taszakowski, **sp. n.**, are described from Sulawesi, Indonesia. Photographs of the male and female habitus and male genitalia are presented and a short comparison with morphologically similar genera is drawn.

## Introduction

The Isometopinae are one of the least numerous and poorly known subfamilies within Miridae. The group has a worldwide distribution (Schuh 2002–2013, Casis and Schuh 2012, Cassis 2016) but, due to a cryptic habitus, the representatives are relatively rare in collections. Forty-three genera and 249 species have been described in this most thoroughly studied subfamily of Miridae. The most diverse genera are *Isometopus* Fieber and *Myiomma* Puton (Herczek and Popov 2011). An autapomorphic subfamily, the Isometopinae differ from other mirids in possessing paired ocelli between the compound eyes. Previous information clearly indicated that isometopines either inhabit bark, where they feed on scale insects (Wheeler and Henry 1978, Yasunaga and Hayashi 2002), or are predators of soft-bodied insects. Akingbohungbe (1996), Wheeler (2001) and Yasunaga (2005) reviewed the biology of isometopines.

Schwartz and Schuh (1990) established the new genus *Gigantometopus* and species *G.rossi*, from Sumatra. Akingbohungbe (2012) described *G.schuhi* as a new species from Borneo, and Yasunaga and Hayashi (2002) created a new genus *Astroscopometopus*, which comprises *A.gryllocephalus* (Miyamoto, Yasunaga & Hayashi, 2002) from Japan and *A.formosanus* (Lin, 2005) from Taiwan. All authors recognized a close relationship between the described genera and *Isometopidealieweni* Poppius from Sri Lanka. Lin (2005) described a second species of *Isometopidea*, *I.yangi* from Taiwan. Recently several specimens (one female and eight males) were found in the Heteroptera collection of the Royal Belgian Institute of Natural Sciences (Brussels); they represent a new genus and species that seem closely related to the above-mentioned genera.

## Material and methods

Color photographs were obtained using a Leica M205C (stereomicroscope), Leica DFC495 (camera), and Leica application suite 4.9.0 (software). Photographs were obtained using a Nikon Eclipse E 600 microscope and the computer program NIS Elements, ver. 4.10. Specimens for SEM analysis were prepared using a modified method of Kanturski et al. (2015) and were imaged with the Phenom XL field emission scanning electron microscope and the Hitachi SU8010 field emission scanning electron microscope FESEM.

Measurements were made with a micrometer and are presented in millimeters (mm). Dissections of male genitalia were performed using Kerzhner and Konstantinov’s (1999) technique. The terminology for genital structures follows Konstantinov (2003). The study was based on material deposited in the collection of the Royal Belgian Institute of Natural Sciences (R.I.Sc.N.B).

## Taxonomy

### Genus Sulawesimetopus gen. n.

Taxon classificationAnimaliaHemipteraMiridae

http://zoobank.org/95B19859-C36A-4E19-B457-8A6DFF5487C2

#### Type species.

*Sulawesimetopushenryi* sp. n.

#### Diagnosis.

**Figure 1.**
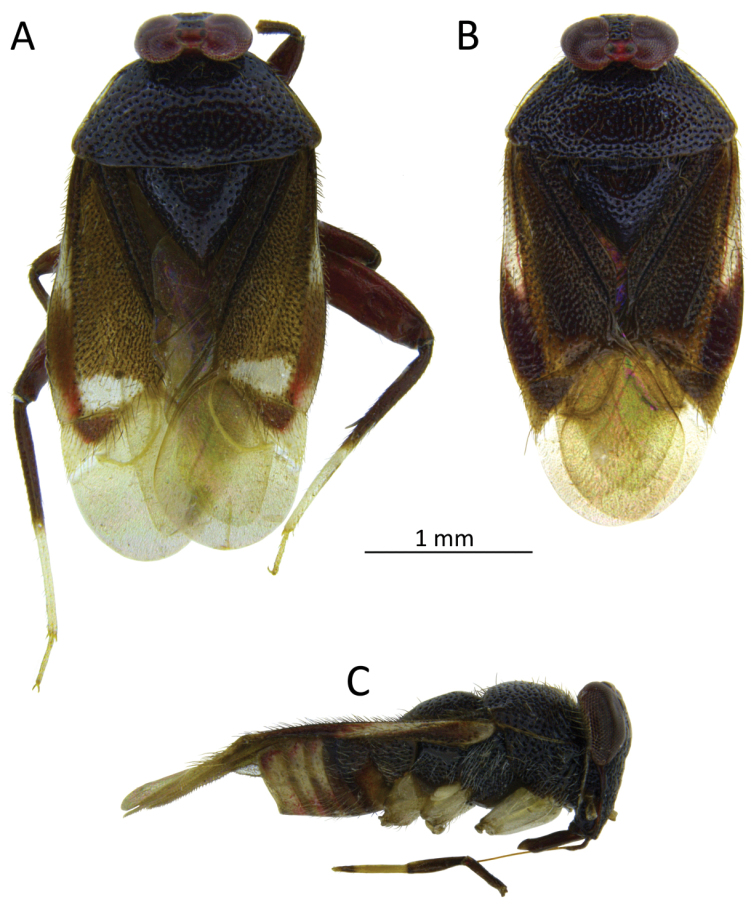
*S.henryi*, female (**A**) and male (**B, C**) dorsal and lateral view.

**Figure 2.**
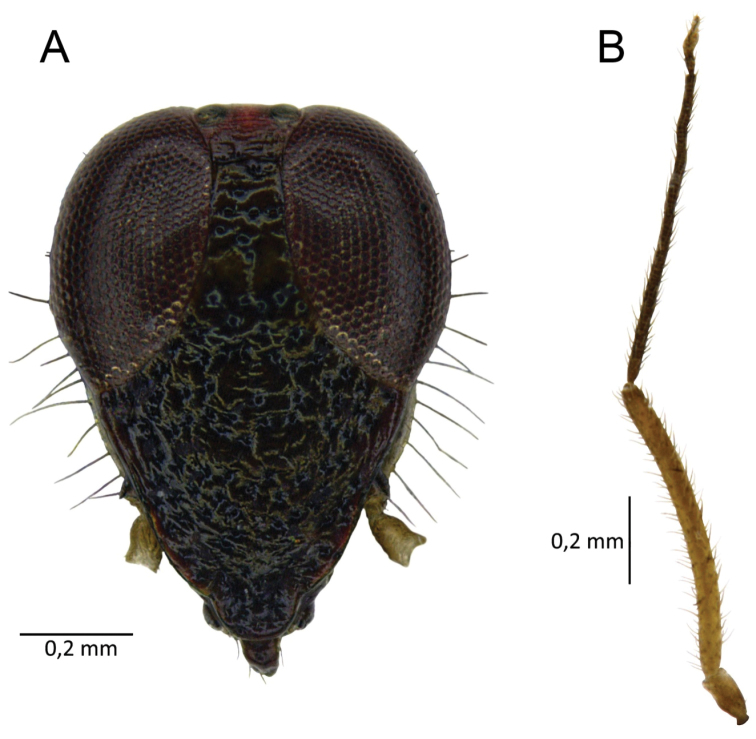
*S.henryi*, male, front of head (**A**), left antenna (**B**).

Dorsum densely and deeply punctuate, with uniformly distributed dark-brown, semierect long setae. Head vertical, flattened in front, almost as high as pronotal disc, covering very narrow collar and very poorly marked calli (partly). Front and lateral parts of head strongly wrinkled and deeply punctuate, lateral edges of head with long, protruding setae (Fig. 2A). Eyes large, nearly at same level as vertex, producing concavity behind it. Fovea antennalis removed from ventral eye margin (Fig. 1A–C). Antennal segments I and II of almost same thickness, III and IV thinner. All segments except 1^st^ with white, adjacent setae of diameter not exceeding segment thickness (Fig. 2B). Labium reaching second abdominal segment. Pronotum with very weakly marked calli, narrow but distinct collar, narrow lateral carina and slightly convex posterior margin. Mesoscutum very narrow, scutellum strongly tumid, sunken basomedially (Fig. 1A, B). Exocorium, pro-, meso- and metapleuron densely and deeply punctuate. Mesofemora with five, metafemora with 6 trichobotria (Fig. 9A–D). All tarsi two-segmented with second segments longer than 1^st^, incompletely divided (Fig. 5). Claws without subapical tooth (Fig. 6 A). Ostiolar peritreme occupying entire lower part of metepisternum and apical part of metafemur (Fig. 8B). Aedeagus delicate, endosoma sacciform and membranous, weakly sclerotized inside (Fig. 7D). Left paramere scythe-shaped, sensory lobe with several long setae; apical process elongated, expanded at middle with several tiny spikes; right paramere short, with knee-shaped sensory lobe, hypophysis with several tiny spikes (Fig. 7B, C).

**Figure 3.**
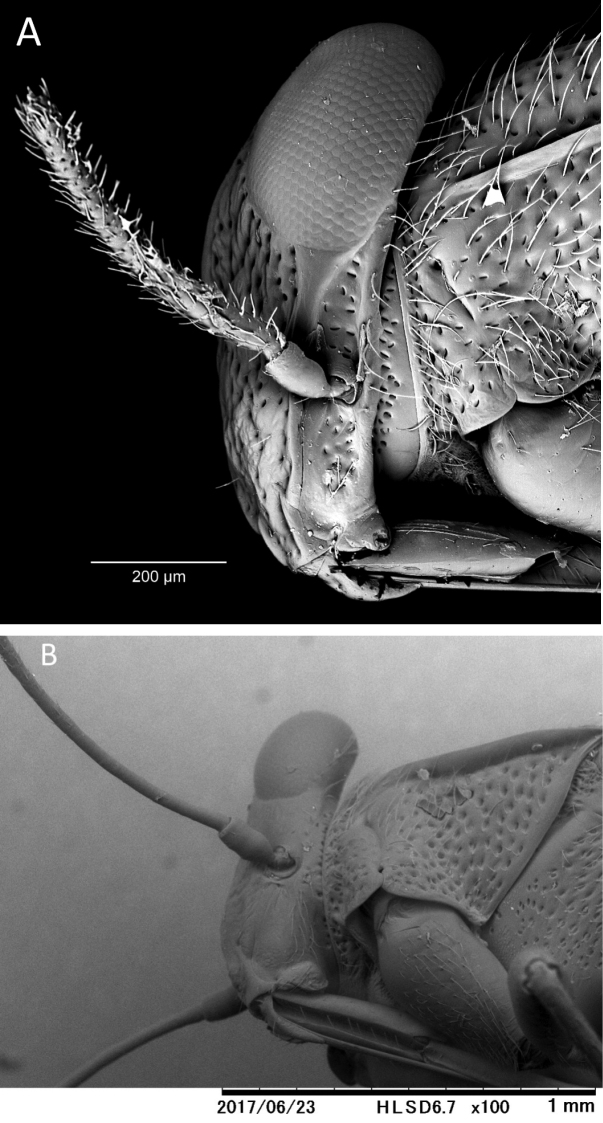
*S.henryi* sp.n., male, head, lateral view (**A**), *Astroscopometopusgryllocephalus*, male, head, lateral view (**B**).

**Figure 4.**
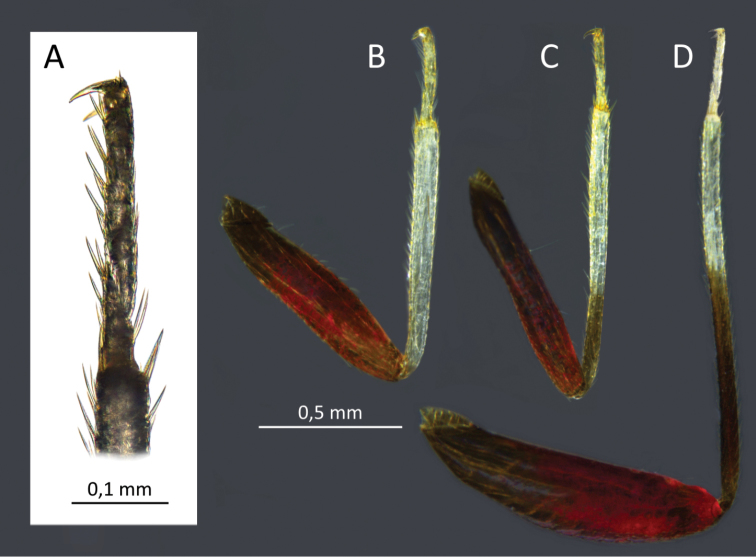
*S.henryi*, male, 1^st^ leg tarsus (**A**), legs: 1^st^ (**B**), 2 ^nd^(**C**), 3^th^(**D**).

**Figure 5.**
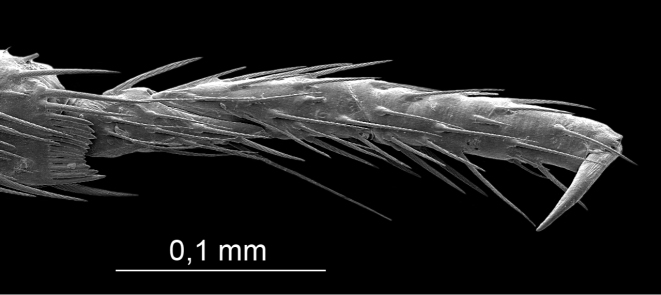
*S.henryi*, male, 1^st^ leg tarsus.

**Figure 6.**
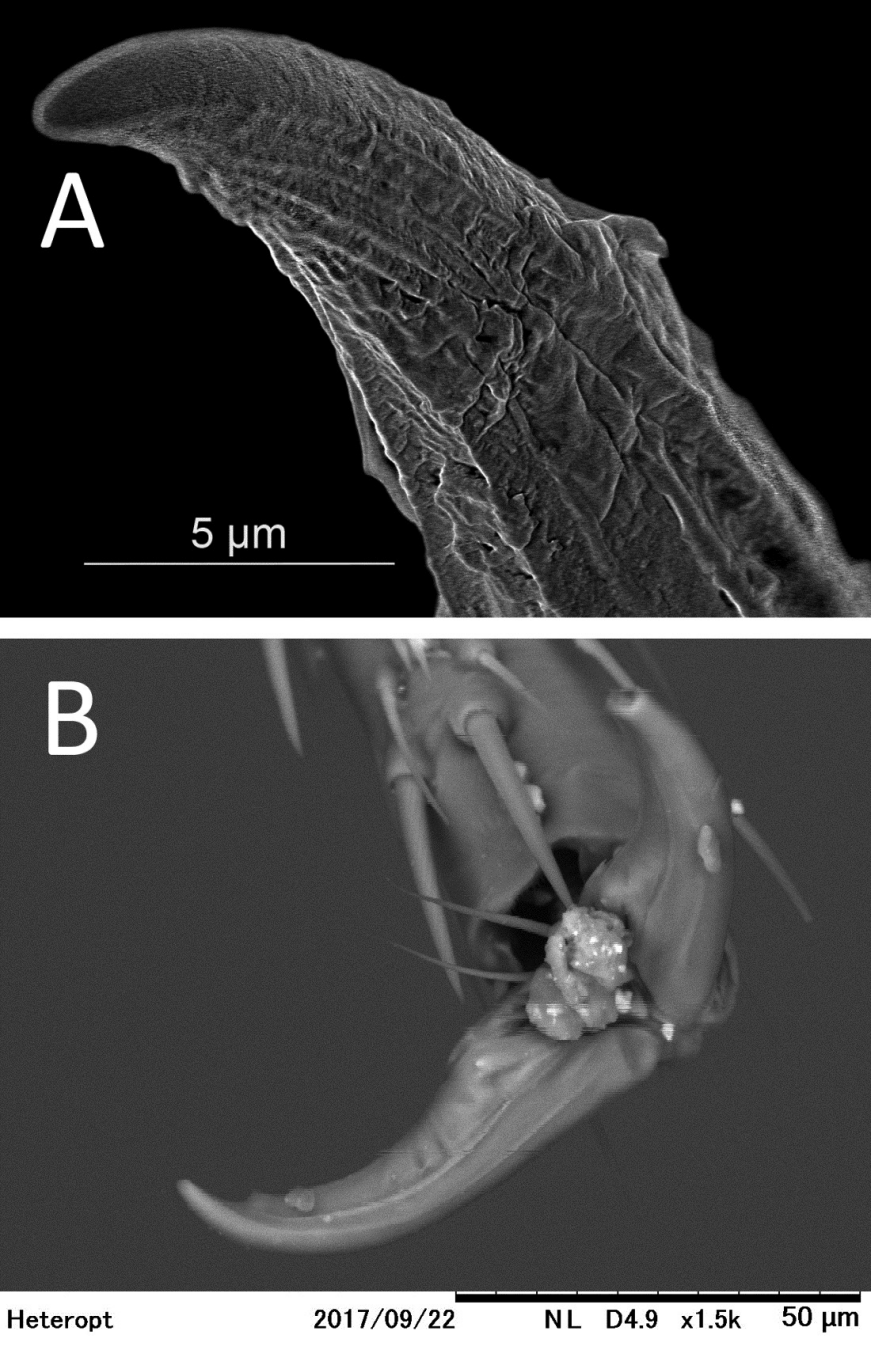
*S.henryi*, male, claw, 2nd leg (**A**), *Astroscopometopusgryllocephalus*, male, claws (**B** images taken by T. Yasunaga, courtesy of CSR Division, Hitachi High –Technologies Corporation, Tokyo).

**Figure 7.**
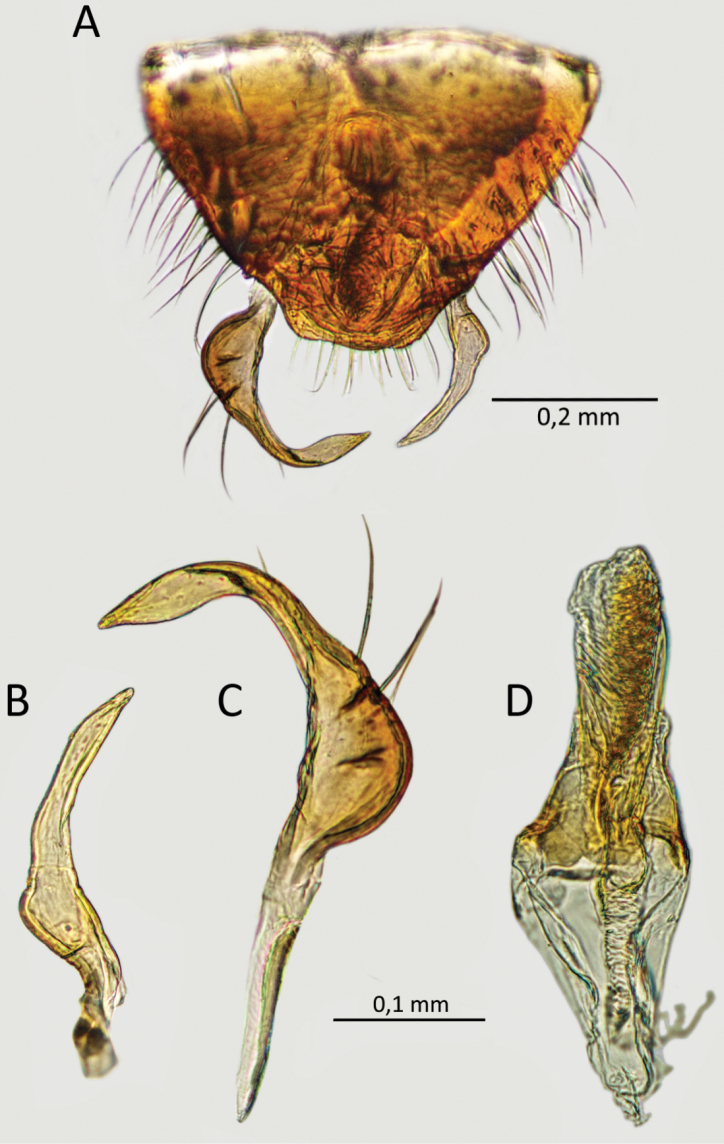
*S.henryi*, male genitalia genital capsule (**A**), right paramere (**B**), left paramere (**C**), phallus (**D**).

**Figure 8.**
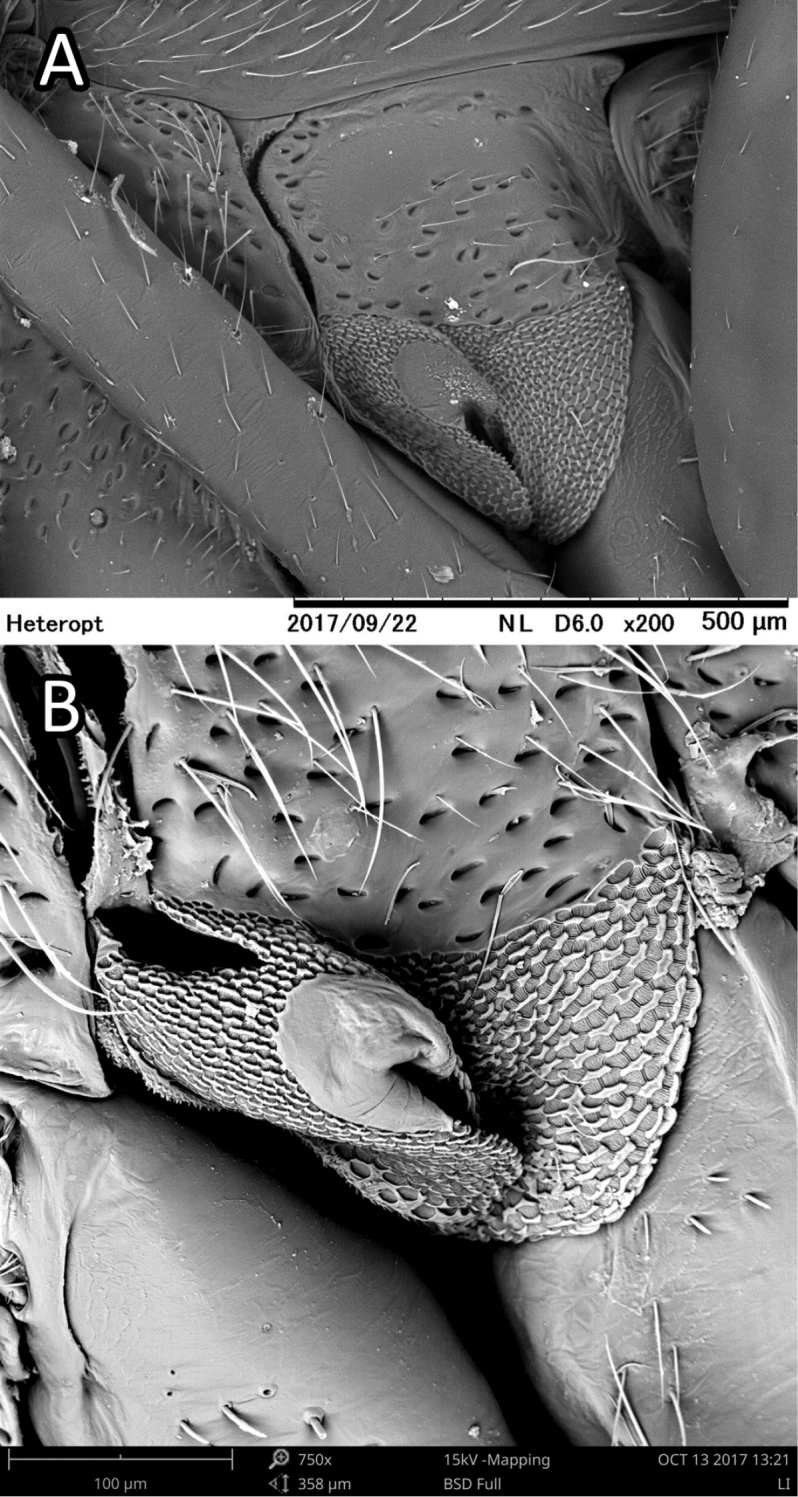
*Astroscopometopusgryllocephalus*, male, ostiolar peritreme (**A** images taken by T. Yasunaga, courtesy of CSR Division, Hitachi Hig Male) **B***S.henryi*, male, ostiolar peritreme.

**Figure 9.**
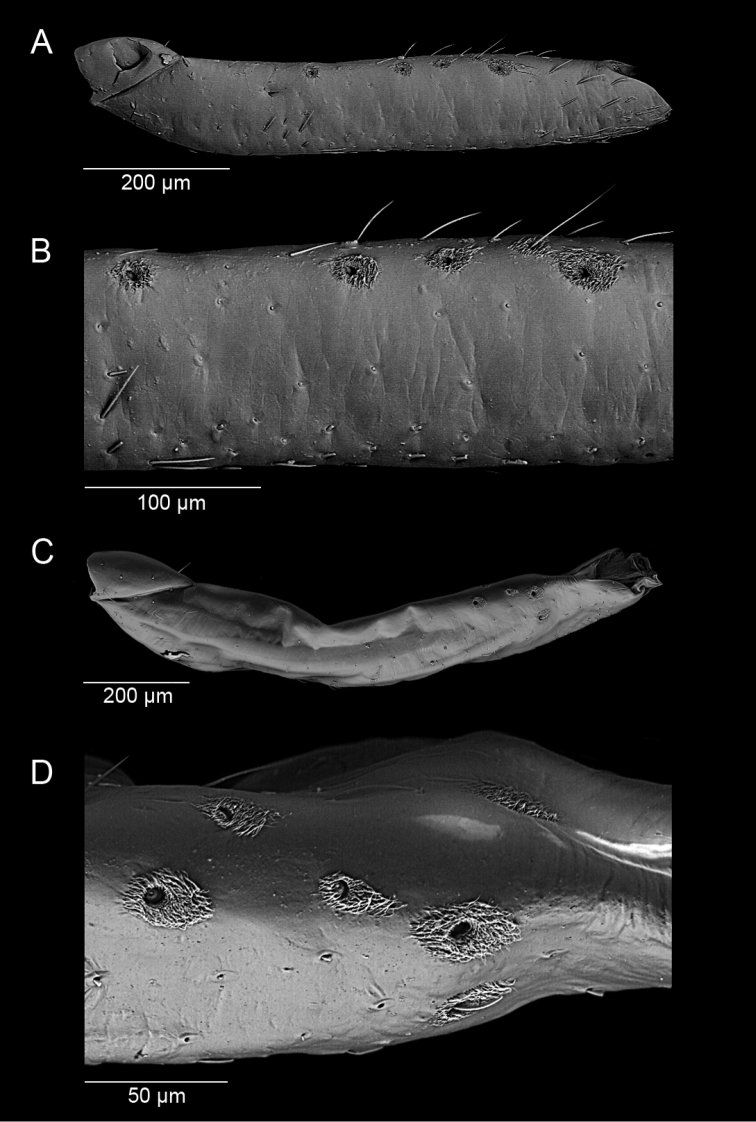
*S.henryi*, male, femoral trichobothria. **A, B** mesofemora **C, D** metafemora.

#### Etymology.

Name combines Sulawesi (the type locality) with part of the generic name *Isometopus*, the type genus of the subfamily.

#### Remarks.

Herczek (1993) established Gigantometopini, one of four tribes belonging to Isometopinae. At that time, only one genus and species had been described: *Gigantometopusrossi* Schwartz & Schuh, 1990. This species is the largest known isometopine (6.98 mm). Distinctive features of this tribe include the size of body, distinct calli separated by a deep incision, a strongly swollen scutellum, a well-marked 1A on the clavus, 5 and 6 meso- and metafemoral trichobothria, 3-segmented tarsi and claws without a subapical tooth. *Gigantometopusschuhi* from Borneo, described by Akingbohungbe in 2012, is significantly smaller than *G.rossi*, but other features allow it to be placed in this genus. We agree with Akingbohungbe’s (2012) opinion that the large size is peculiar to the nominotypical species but not to the genus. Additionally, the genus *Astroscopometopus*, described by Yasunaga and Hayashi (2002), has features similar to those of *Gigantometopus* Schwartz and Schuh, 1990 and *Isometopidea* Poppius, 1913. Also *Sulawesimetopus* resembles the genera *Gigatometopus* and *Isometopidea*, but differs from them in several basic features including deep and densely punctured dorsum and thorax pleurites, pronotum with slightly convex posterior margin and narrow lateral carina, extremely reduced calli, and the lack of a middle fossa. Other differences include a very narrow (or lack of) mesoscutum and an indistinct division of the 2^nd^ and 3^rd^ tarsomeres. In addition, *Sulawesimetopus* differs from *Isometopidea* Poppius by the shape of the head, placement of fovea antennalis, shorter claval commissure and shorter cuneus. These species, however, share numerous femoral trichobothria. Such a combination of characters allows the new genus and species to be assigned to Gigantometopini. However, as has been done by Yasunaga et al. (2016), it is necessary to revise the suprageneric classification of the Isometopinae.

### Sulawesimetopus henryi sp. n.

Taxon classificationAnimaliaHemipteraMiridae

http://zoobank.org/45CD9418-019C-4790-940B-B095762D4E38

#### Diagnosis.

Same as genus.

#### Etymology.

Named in honor of the well-known American hemipterologist Dr. Thomas J. Henry, who has made a great contribution to the study of Miridae.

#### Description.

Male. *Coloration* (Fig. 1A–C): body mostly shiny, dark brown. *Head*: dark brown, 1.34 in male and 1.47 in female, as high as wide and respectively 0.51 and 0.47 of pronotal width; eyes brownish red, the area around ocelli reddish. Antennae thin (particularly segments III and IV), I and II yellowish, III brown, IV yellow brown (Fig. 2B). Labium shiny, first three segments brown, IV with a dark ring in distal part (Fig. 1C). *Thorax*: pronotum chocolate brown, lateral edges clear, transparent and slightly raised. Mesoscutum very narrow, blackish brown, scutellum chocolate brown, excavated mesally, 0.80 as long as wide. Propleuron, mesopleuron and metapleuron dark brown. Claval commissure distinct, 0.46 as long as length of scutellum. *Abdomen*: bicolored: four segments before genital one lighter, yellowish tinged with pink, others dark brown. Ostiolar peritreme ivory, evaporative area brown (Figs 1C, 8, 9). *Hemelytron*: in various shades of brown, a bit lighter than pronotum and scutellum. Median part of embolium with elongate white spot. Lateral and apical part of cuneus and part of medial fracture adjacent to cuneus yellowish brown. Cuneus as long as wide, yellowish brown, central part dark brown. Membrane pale grey, semitransparent, with two yellowish-brown cells (the small one barely visible). *Legs*: coxae pale, almost white, femora and basal ½ tibiae chestnut-brown. Distal part of tibiae and tarsi almost white. Metacoxae flattened and thickened (Fig. 4D).

Female. Larger, head and pronotum similar to male in coloration, structure and texture. Mesoscutum covered by pronotum, invisible. Corium yellowish brown, median part of exocorium and part of corium adjacent to cuneus and most of cuneus white (Fig. 1A). Distal part of embolium near cuneal fracture red tinged. Membrane with creamy veins. Second tarsal segments incompletely divided.

Measurements. Holotype, male (number of measured specimens and range of variation in parentheses): body length: 3.10 (n = 6: 3.05–3.25), width: 1.47 (n = 6: 1.37–1.52); head length: 0.27 (n = 8: 0.27–0.30), width: 0.71 (n = 8: 0.68–0.74), height: 0.95 (n = 8: 0.92–1.03); dorsal width of eye: 0.30 (n = 8: 0.28–0.32); vertex width: 0.19 (n = 8: 0.19–0.21); antennal segments: I–0.13 (n = 8: 0.11–0.13), II–0.71 (n = 7: 0.65–0.72), III–0.78 (n = 8: 0.8–0.78), IV–0.13 (n = 7: 0.19–0.13); rostral segments: I–0.35 (n = 3: 0.38–0.35), II–0.45 (n = 3: 0.45–0.50), III–0.33 (n = 2: 0.33–0.38), IV–0.39 (n = 2: 0.39–0.50); pronotum length: 0.57 (n = 8: 0.57–0.62), anterior width: 0.74 (n = 8: 0.68–0.77), posterior width: 1.37 (n = 8: 1.32–1.46); mesoscutum length: 0.03 (n = 8: 0.02–0.04); scutellum length: 0.69 (n = 8: 0.61–0.72), width:0.81 (n = 8: 0.79–0.83) ; claval commissure length: 0.37 (n = 7: 0.28–0.37); hind leg: femur length: 1.05 (n = 2: 1.05–1.17), width: 0.30; tibia length: 1.40 (n = 3: 1.30-1.45), tarsus: 0.30 (n = 3: 0.25–0.30) I-0.11, II-0.25 (supposedly two segments 0.11+0.13); cuneus length: 0.36 (n = 8: 0.34–0.37), width: 0.35 (n = 8: 0.34–0.36).

Female (one specimen): body length: 3.25, width: 1.55; head length: 0.25, width: 0.70, height: 1.03; dorsal width of eye: 0.29; vertex width: 0.20; antennal segments: I–0.12, II–0.65, III–0.87, IV–0.18; rostral segments: invisible; pronotum length: 0.67, anterior width: 0.88, posterior width: 1.50; scutellum length: 0.65; claval commissure length: 0.38; hind leg: femur length: 1.13, width: 0.35; tibia length: 1.45, tarsus: 0.35 (I–0.07, II+III–0.30) ; cuneus length: 0.36; width: 0.36

Material examined. Holotype: male. Indonesia, Sulawesi Utara, P.P.R. bungalow (P.M.), 8/18 XI 1985, Station: 099, Project Wallace, leg: R. Bosmans & J. Van Stalle.

Female: Sulawesi Utara, Dumoga-Bone Nat.Park, Hogg’sBack subcamp (660m), 15-XI-1985. Station: 095. Project Wallace, leg: R. Bosmans & J. Van Stalle. L.G. n^o^ 26.977.

Paratypes: : 2 ♂♂. Indonesia, Sulawesi Utara, P.P.R. bungalow (P.M.), 8/18 XI 1985, Station: 099, Project Wallace, leg: R. Bosmans & J. Van Stalle; 5♂♂ Sulawesi Utara, Dumoga-Bone Nat.Park, Hogg’sBack subcamp (660m), 15-XI-1985. Station: 095. Project Wallace, leg: R. Bosmans & J. Van Stalle. L.G. n^o^ 26.977. The holotype and paratypes are deposited in the R.I.Sc.N.B.

**Remarks.** The new species can be distinguished from all others belonging to *Gigantometopus*, *Astroscopometopus* and *Isometopidea* by its body structure, combination of color, and metric features. The newly described species is the smallest of those in the three genera (Table 1). The following metric features distinguish *Sulawesimetopushenryi* sp.n.: head width to vertex width ratio 3.55 (vs. *Gigantometopusschuhi* 2.05, *Astroscopometopusgryllocephalus* 3.11, *A.formosanus* 3.25 and *Isometopideayangi* 7.0), head width to pronotum width 0.51 (and respectively 0.28, 0.45, 0.46 and 0.54), antennal segments II:I length ratio 5.96 (and respectively 6.5, 7.67, 6.50 and 7.5), pronotum width to head width 1.94 (and respectively 3.51, 2.24, 2.15 and 1.85). In the new species the claval commissure is shorter than in others. Additionally *S.henryi* sp.n. differs from *A.gryllocephalus* by position of the scutellar depression, antennal hairs (Fig. 3A, B) and the lack of a subapical claw tooth. Certain color features also differ: *S.henryi* sp.n. is darker than the others, the apical part of the rostrum is dark brown, the hemelytra are almost monochromatic (dark brown), and only the middle part of the embolium is white. The hemelytra in *G.schuhi* are largely dark golden to reddish brown, in contrast to the hemelytra light brown with a creamy spot in the middle in *G.gryllocephalus*, grey with a dark brown clavus and a circular creamy spot mesially in *A.formosanus* and the yellowish brown semitransparent hemelytra in *Isometopideayangi*. The color pattern of the legs also is species–specific.

**Table 1.** Comparison of metric features of known species of *Sulawesimetopus*, *Gigantometopus*, *Astroscopometopus* and *Isometopidea*.

Measurement	*S.henryi* sp. n.		* G. schuhi *	* G. rossi *	* A. gryllocephalus *		* A. formosanus *	* I. yangi *
	♂^*^	♀	♂	♀	♂	♀	♂	♂
Body length	3.10	3.25	3.28	6.98	3.6	4.06	4.1	4.2
Body width	1.48	1.55	1.44	1.49	1.50	1.49	1.6	1.7
Head length	0.28	0.25	0.27	?	?	0.23	0.30	0.30
Head width	0.71	0.70	0.41	1.03	0.59	0.62	0.65	0.70
Head height	0.95	1.03	1.06	1.71	0.97	0.99	1.1	1.0
Dorsal width of eye	0.31	0.29	0.13	0.35	0.20	0.23	0.23	0.30
Vertex width	0.20	0.20	0.20	0.32	0.19	0.16	0.20	0.10
Antennal segments I:II:III:IV	0.12:0.72: :0.78:0.13	0.12:0.6: 0.87:0.18	0.14:0.91: 0.93:0.20	0.23:1.64: 1.10:0.9	0.15:1.18: 0.67:0.29	0.16:1.00: 0.78:0.25	0.20: 1.30:-:-	0.2:1.5 :0.3:0.2
Rostral segments I:II:III:IV	1.5 (0.35:0.45 :0.33:0.39)	invisible	1.84	3.10 0.88:-:-:-	?	1.89	2.0	2.0
Pronotum length	0.59	0.67	0.60	1.55	0.69	0.63	0.90	0.6
Posterior width of pronotum	1.38	1.50	1.44	2.69	1.32	1.38	1.40	1.3
Scutellum length	0.69	0.65	0.64	?	?	0.69	0.60	0.6
Scutellum width	0.81	0.77	0.69	?	?	0.37	0.7	0.6
Claval commissure	0.32	0.38	0.30(?)	?	?	?	0.4	0.5
Hind femur length	1.11	1.13	?	?	1.14	1.26	?	?
Hind tibia length	1.43	1.45	?	?	1.81	1.90	?	?
Tarsus length	0.30	0.35	?	?	0.36	0.34	?	?
Tarsal segments length I:II:III	0.11:0.25 (0.11:0.13)	0.07:0.30	?	?	?	0.13:0.16 :0.19	0.60	?
Cuneus length	0.36	0.35	0.53	0.78	?	?	0.6	0.7
Cuneus width	0.36	0.35	0.31	?	?	?	?	0.3

* averaged values are given

The construction of the aedeagus and parameres is similar to other compared species. The differences are relatively small (as with most other species of Isometopinae and Psallopinae) and refer to the extent of sclerotization of the aedeagus and the shape of the sensory lobe of the left paramere.

The female of *S.henryi* sp. n. is indistinguishable from a female of *A.gryllocephalus* by the length of antennal segments, posterior width of the pronotum, and the shorter hind femur, tibia and tarsus. The proportions of body length to width, head width to vertex width, and corium length to cuneus length also differ (respectively: 2.09 and 2.72, 3.50 and 3.88, 6.94 and 4.11). Clear differences occur in coloration. The distal part of the embolium near the cuneal fracture in *S.henryi* sp. n. is tinged with red, the median part of exocorium adjacent to the cuneus is yellowish brown and the cuneus is mostly white, whereas in *A.gryllocephalus* the embolium is pale brown and semitransparent, the corium is yellowish with a white spot in the middle, and the inner half of the cuneus is yellowish white.

## Supplementary Material

XML Treatment for Sulawesimetopus

XML Treatment for Sulawesimetopus henryi

## References

[B1] Akingbohungbe AE (1996) The Isometopinae (Heteroptera: Miridae) of Africa, Europe, and the Middle East. Delar Tertiary Publishers, Ibadan, 170 pp.

[B2] Akingbohungbe AE (2012) A note on *Gigatometopus* Schwartz and Schuh (Heteroptera: Miridae: Isometopinae) with the description of a new species from Borneo. Entomologica Americana 118(1/4): 130–132. 10.1664/12-RA-015.1

[B3] Cassis G Schuh RT (2012) Systematics, biodiversity, biogeography, and host associations of the Miridae (Insecta: Hemiptera: Heteroptera: Cimicomorpha). Annual Review of Entomology 57: 377–404. 10.1146/annurev-ento-121510-133533 22149267

[B4] Cassis G (2016) Review of the seven new species of Isometopinae (Heteroptera: Miridae) in Australia and discussion of distribution and host plant associations of the subfamily on a worldwide basis. Austral Entomology 55: 392–422. 10.1111/aen.12202

[B5] Herczek A (1993) Systematic position of Isometopinae Fieb. (Miridae, Heteroptera) and their intrarelationships. Prace Naukowe Uniwersytetu Śląskiego, Katowice, 1357: 1–86.

[B6] Herczek A Popov YA (2011) New Isometopinae (Hemiptera: Heteroptera: Miridae) from the Oriental Region, with some notes on the genera *Alcecoris* and *Sophianus*. Zootaxa 3023: 43–50.

[B7] Kanturski M Karcz J Wieczorek K (2015) Morphology of the European species of the aphid genus *Eulachnus* (Hemiptera: Aphididae: Lachninae) – a SEM comparative and integrative study. Micron 76: 23–36. 10.1016/j.micron.2015.05.004 26021259

[B8] Kerzhner IM Konstantinov FV (1999) Structure of the aedeagus inMiridae (Heteroptera) and its bearing to suprageneric classification. Acta Societatis Zoologicae Bohemicae 63: 117–137.

[B9] Konstantinov FV (2003) Male genitalia inMiridae (Heteroptera) and their significance for suprageneric classification of the family. Part I: general review, Isometopinae and Psallopinae. Belgian Journal of Entomology 5: 3–36.

[B10] Lin CS (2004) Seven new species of Isometopinae (Hemiptera: Miridae) from Taiwan. Formosan Entomologist 24: 317–326.

[B11] Lin CS (2005) New or little-known Isometopinae from Taiwan (Hemiptera: Miridae). Formosan Entomologist 25: 195–201.

[B12] Miyamoto S Yasunaga T Hayashi M (1996) Description of a new isometopine plant bug, Isometopidea gryllocephala, found on Ishigaki Island, Japan (Insecta, Heteroptera, Miridae). Species Diversity 1: 107–110. 10.12782/specdiv.1.107

[B13] Schuh RT (2002–2013) On-line systematic catalog of plant bugs (Insecta: Heteroptera: Miridae). http://research.amnh.org/pbi/catalog [Accessed 10August 2015]

[B14] Wheeler AG Jr Henry TJ (1978) Isometopinae (Hemiptera: Miridae) in Pennsylvania: biology and descriptions of fifth instars, with observations of predation on obscure scale. Annals of the Entomological Society of America 71: 607–614. 10.1093/aesa/71.4.607

[B15] Wheeler AG (2001) Biology of the plant bugs (Hemiptera: Miridae): Pests, Predators, Opportunists. Cornell University Press, Ithaca.

[B16] Yasunaga T Hayashi M (2002) New or little known isometopine plant bugs from Japan (Heteroptera: Miridae). Tijdschrift voor Entomologie 145: 95–101. 10.1163/22119434-900000103

[B17] Yasunaga T (2005) Isometopinae plant bugs (Heteroptera: Miridae) preferably inhabiting *Fraxinusgriffithii* on Ishigaki Island of the Ryukyus, Japan. Tijdschrift voor Entomologie 148: 341–349. 10.1163/22119434-900000179

[B18] Yasunaga T Duangthisan J Yamada K Artchawakom T (2016) Further records of the plant bug subfamily Isometopinae from Thailand (Heteroptera: Miridae) with description of three new species. Tijdschrift voor Entomologie 159: 89–96. 10.1163/22119434-15902003

